# Remarks upon a Cause of Error, in Estimating the Degree of Sensibility in Affections of the Nerve-Centres, and, Particularly of the Posterior Columns of the Spinal Cord

**Published:** 1870-03

**Authors:** 


### REMARKS UPON A CAUSE OF ERROR, IN ESTIMATING THE DEGREE OF SENSIBILITY IN AFFECTIONS OF THE NERVE-CENTRES, AND, PARTICULARLY OF THE POSTERIOR COLUMNS OF THE SPINAL CORD.

BY DR. BROWN-SÉQUARD.

Translated for the EXAMINER, from *Archives de Physiologie et Normale et Pathologique.*

The cause of error which I shall describe does not seem to be understood. It is, nevertheless, so frequently committed, that I think a delineation of it will be useful. Let us suppose, taking an example that will be comprehended more easily, perhaps, than any other, that one is about to examine a patient, with induration of one posterior column, occupying the entire extent of the dorso-lumbar region. The individual has ataxia of the abdominal member of the injured side. It is observed in studying the sensibility of this part, that the two points of the æsthesiometer are felt, the differences of temperature are perceived, and that tickling and painful sensations are resented, as much, or nearly as much as in the normal state. It is, therefore, concluded, that the different kinds of sensibility exist in him, without any alteration. This is conveying a great mistake; *indeed, the fact that the sensibility seems to be in the normal state, that is to say, is not augmented, suffices to show that it is in reality diminished in an apparent degree.*

I will explain myself—the induration of the posterior columns, like any other lesion of the cord, is a potent cause of hyperæsthesia, or the augmentation of the different kinds of sensibility (touch, tickling, pain, and temperature). If, then, the patient does not have *increased* and *keener* sensations of the different impressions made on the ataxic member, it is because he is affected by anæsthesia, in the same parts that are hyper-

æsthetic. If this sensibility seems to have preserved its normal degree, it is on account of this faculty losing all that it should have gained, if there had not been a cause of anæsthesia coëxisting with the cause of hyperæsthesia. It is, then, a mistake to say, the sensibility is normal, where it seems to be so only, and where it is known it ought to be augmented.

In all cases of affection of the nerve-centres, where the location of the injury is indicated, and where it is known by physiological data, or from the pathology, that hyperæsthesia should exist, if it is not found, we must conclude that anæsthesia exists, even when the different kinds of sensibility are in their normal degree; and, we must admit, that the two morbid alterations (anæsthesia and hyperæsthesia) compensate each other.*

* This localization of the induration is rare; I have only observed it in three cases. An error analogous to the preceding is frequently committed, in regard to the voluntary movement, in those who have ataxia, from induration of the posterior columns. Some involuntary spasmodic movements often combine themselves with the voluntary movements, giving them the appearance of a power, as great, and even greater, than that of the normal state; consequently, it is affirmed, there is no paralysis, when it really exists, and, frequently, in an appreciable degree.

